# Implementation of Basal–Bolus Therapy in Type 2 Diabetes: A Randomized Controlled Trial Comparing Bolus Insulin Delivery Using an Insulin Patch with an Insulin Pen

**DOI:** 10.1089/dia.2018.0298

**Published:** 2019-05-07

**Authors:** Richard M. Bergenstal, Mark Peyrot, Darlene M. Dreon, Vanita R. Aroda, Timothy S. Bailey, Ronald L. Brazg, Juan P. Frias, Mary L. Johnson, David C. Klonoff, Davida F. Kruger, Shenaz Ramtoola, Julio Rosenstock, Pierre Serusclat, Ruth S. Weinstock, Ramachandra G. Naik, David M. Shearer, Vivien Zraick, Brian L. Levy

**Affiliations:** ^1^ International Diabetes Center, Park Nicollet, Minneapolis, Minnesota.; ^2^ Loyola University Maryland, Department of Sociology, Baltimore, Maryland.; ^3^ Calibra Medical, Johnson & Johnson Diabetes Care Companies, Wayne, Pennsylvania.; ^4^ Medstar Health Research Institute, Hyattsville, Maryland.; ^5^ AMCR Institute, Inc., Escondido, California.; ^6^ Rainier Clinical Research Center, Renton, Washington.; ^7^ National Research Institute, Los Angeles, California.; ^8^ Diabetes Research Institute, Mills-Peninsula Medical Center, San Mateo, California.; ^9^ Division of Endocrinology, Diabetes, Bone and Mineral Disease, Henry Ford Health System, Detroit, Michigan.; ^10^ East Lancashire Hospitals NHS Trust, Blackburn, United Kingdom.; ^11^ Dallas Diabetes Research Center at Medical City, Dallas, Texas.; ^12^ Groupe Hospitalier Mutualiste Les Portes du Sud, Vénissieux, France.; ^13^ SUNY Upstate Medical University, Department of Endocrinology, Diabetes and Metabolism, Syracuse, New York.

**Keywords:** Mealtime insulin patch, Type 2 diabetes

## Abstract

***Background:*** Barriers to mealtime insulin include complexity, fear of injections, and lifestyle interference. This multicenter, randomized controlled trial evaluated efficacy, safety, and self-reported outcomes in adults with type 2 diabetes, inadequately controlled on basal insulin, initiating and managing mealtime insulin with a wearable patch versus an insulin pen.

***Methods:*** Adults with type 2 diabetes (*n* = 278, age: 59.2 ± 8.9 years), were randomized to patch (*n* = 139) versus pen (*n* = 139) for 48 weeks, with crossover at week 44. Baseline insulin was divided 1:1 basal: bolus. Using a pattern-control logbook, subjects adjusted basal and bolus insulin weekly using fasting and premeal glucose targets.

***Results:*** Glycated hemoglobin (HbA1c) change (least squares mean ± standard error) from baseline to week 24 (primary endpoint) improved (*P* < 0.0001) in both arms, −1.7% ± 0.1% and −1.6% ± 0.1% for patch and pen (−18.6 ± 1.1 and −17.5 ± 1.1 mmol/mol), and was maintained at 44 weeks. The coefficient of variation of 7-point self-monitoring blood glucose decreased more (*P* = 0.02) from baseline to week 44 for patch versus pen. There were no differences in adverse events, including hypoglycemia (three severe episodes per arm), and changes in weight and insulin doses. Subject-reported treatment satisfaction, quality of life, experience ratings at week 24, and device preferences at week 48 significantly favored the patch. Most health care providers preferred patch for mealtime insulin.

***Conclusions:*** Bolus insulin delivered by patch and pen using an algorithm-based weekly insulin dose titration significantly improved HbA1c in adults with type 2 diabetes, with improved subject and health care provider experience and preference for the patch.

## Introduction

Type 2 diabetes is a rapidly growing epidemic, challenging health care providers and health care systems to manage its acute and long-term effects.^1,2^ Despite an increasing number of treatment options, only about 50% of people with diabetes on any therapy and <30% of people with diabetes using some form of insulin therapy achieve the recommended treatment goal of glycated hemoglobin (HbA1c) <7.0% (<53 mmol/mol).^3–5^ Current guidelines recommend a stepwise approach to treatment intensification, with a combination of oral and/or injectable antihyperglycemic agents for people with type 2 diabetes who do not achieve glycemic goals with lifestyle management and pharmacologic monotherapy (typically metformin).

Insulin therapy usually is initiated with a basal formulation, which primarily targets control of fasting plasma glucose. Options for further intensification of injected therapy include the following: adding rapid-acting mealtime insulin at the largest meal of the day (a “basal–plus” regimen) and, if necessary, then adding mealtime insulin doses at other meals (a “basal–bolus” regimen); adding a glucagon-like peptide (GLP)-1 receptor agonist; or switching to 2 (and if necessary 3) injections of premixed insulin.^3,6–8^

The benefits of early insulin intervention for achieving improved glycemic control are well-established^9^; however, initiation of basal insulin often is delayed by up to 7 years or more.^10^ Similarly, advancement from basal insulin alone to mealtime insulin or other combinations (e.g., GLP-1 receptor agonist therapy) was postponed for an estimated 4.3 years in one database analysis.^11^ This reluctance to intensify treatment is referred to as clinical or therapeutic inertia.^12^ Health care providers may delay treatment intensification because they lack the time and resources to adequately educate the patient and/or they do not have sufficient experience/expertise to implement more complex insulin treatment regimens.^13–15^ Patient barriers to insulin use include fear of injections, perceived social stigma, interference with daily activities, reduced quality of life, and increased cost.^16–18^

Some technological advances to simplify insulin delivery may address some of the abovementioned barriers. In recent years, a number of wearable devices have been designed and developed to deliver basal and/or mealtime insulin.^19^ One such device is the insulin patch or simply “patch” (PAQ MEAL™; CeQur, Marlborough, MA, formerly of Calibra Medical, Wayne, PA), a simplified on-demand subcutaneous delivery device for mealtime insulin that, unlike conventional pumps, is entirely mechanical and not managed by external controllers. Unlike insulin pens, the patch does not require observation of an injection.

The aim of this study was to compare the efficacy and safety of the patch with that of a standard insulin pen for initiating and managing mealtime analog insulin in people with type 2 diabetes not yet achieving the glycemic goal with basal insulin with/without other antihyperglycemic agents. A simple basal–bolus dosing algorithm was implemented. The study also evaluated subject and health care provider preferences for patch versus pen.

## Research Design and Methods

### Study devices

**FIG. 1.**
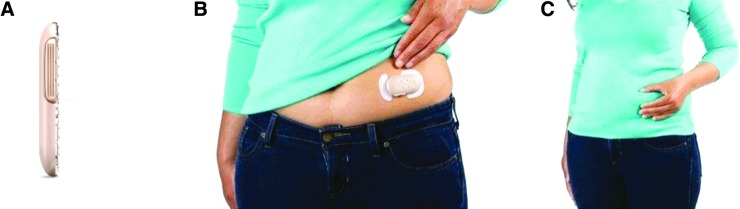
The patch. The wearable, on-demand, mealtime insulin-delivery system patch (Calibra Medical, Wayne, PA) contains up to 200 units of insulin and measures no more than 65 × 35 × 8 mm **(A)**. It can be worn on the abdomen for up to 3 days **(B)**. Mealtime insulin can be dosed in 2-unit increments through clothing by actuating the buttons on both sides of the patch **(C)**.

The patch is a small, wearable mechanical pump (65 × 35 × 8 mm; 10 g before filling) that can be worn on the body for up to 3 days for the delivery of mealtime insulin. In the United States, it is approved for use with rapid-acting insulins lispro (Humalog^®^; Eli Lilly and Co., Indianapolis, IN) and aspart (NovoLog^®^/NovoRapid^®^; Novo Nordisk, Inc., Plainsboro, NJ); aspart was used in the present study. The patch holds up to 200 units of insulin and delivers a 2-unit dose via a subcutaneous cannula with each simultaneous depression of the two buttons on either side of the device. Patients adhere the patch to a cleaned area (100 × 150 mm) on the abdomen. The patch can be worn under clothing and access to the 2 buttons can be achieved either directly or through clothing (Fig. 1).^20^ The comparator device was a NovoLog/NovoRapid FlexPen^®^ (insulin aspart) (Novo Nordisk Pharmaceuticals, Inc., Princeton, NJ).

### Study design

This randomized, multicenter, open-label, parallel, two-arm interventional study (NCT02542631; EudraCT 2015-003761-28) compared efficacy, safety, and self-reported outcomes in adults with type 2 diabetes on basal insulin therapy who initiated and managed mealtime insulin therapy with patch versus pen. The study was conducted in accordance with the Declaration of Helsinki (1964), including all amendments, the International Conference on Harmonization Good Clinical Practice guidelines, International Organization for Standardization regulations, and relevant local laws and regulations. All subjects provided written informed consent before study participation.

Study subjects (22–75 years) were recruited from a total of 62 clinical centers in the following countries: the United States (*n* = 37), France (*n* = 9), Germany (*n* = 6), and the United Kingdom (*n* = 10). Inclusion criteria included a clinical diagnosis of type 2 diabetes, treatment with basal insulin for at least 6 months (with/without antihyperglycemic agents), with a stable dose [≥0.3 units/(kg·day); ≤100 units/day] maintained for at least 6 weeks; HbA1c level of 7.5%–11.0% (53–97 mmol/mol); willingness to perform self-monitoring of blood glucose (SMBG); and a body mass index (BMI) ≤40 kg/m^2^. Exclusion criteria included treatment with mealtime insulin or continuous subcutaneous insulin infusion within the prior year outside of an acute illness or hospital setting; two or more severe hypoglycemic episodes within the prior year; hypoglycemic unawareness; or moderate-to-severe illness.

Following a 4-week screening and baseline period, subjects were randomized according to study site and study device (1:1 to patch or pen). They were followed for 44 weeks to evaluate glycemic control, safety, and treatment experience. At week 44, subjects crossed over to the other treatment arm for 4 weeks to evaluate their preferences for patch versus pen at week 48.

Health care providers completed an experience survey after the last subject at their site finished week 24 of the study.

### Intervention

At randomization, health care providers instructed all subjects to continue taking basal insulin either before their evening meal or at bedtime using insulin glargine (SoloSTAR^®^ pen); those using another basal insulin were switched. Subjects were instructed on the use of their assigned device (patch or pen) for mealtime insulin delivery. Training in the use of the patch included filling the device, applying it to the skin (lower abdomen), and locating the two buttons over clothing to activate dosing. Study-site personnel received patch training and technical support from the study sponsor (CeQur, formerly of Calibra Medical). Blood glucose meters (Verio IQ) for SMBG were provided by LifeScan, Inc. (Wayne, PA).

Concomitant biguanides, alpha-glucosidase inhibitors, sodium–glucose cotransporter 2 inhibitors, thiazolidinediones, and dipeptidyl-peptidase 4 inhibitors approved for use with mealtime insulin were continued at their current doses. Subjects were required to discontinue concomitant sulfonylureas, meglitinides, bromocriptine, dipeptidyl-peptidase 4 inhibitors, and GLP-1 receptor agonists that were not approved for use with mealtime insulin. The prestudy, total daily basal insulin dose was divided 1:1 between basal and mealtime insulin, that is, half of the total daily insulin dose was given as basal insulin and half as mealtime insulin (split evenly between usual daily meals) at randomization. In subjects with HbA1c <9.0% (<75 mmol/mol) at screening, the daily basal insulin dose was reduced by 10% before splitting into basal and mealtime insulins to decrease the potential for hypoglycemia. Subjects were instructed on how to adjust their basal and mealtime insulin doses weekly, using a pattern-based logbook combining SMBG values with a simple insulin adjustment algorithm.^21,22^ No carbohydrate counting was required. Background insulin doses were adjusted each week by adding 2–4 units or subtracting 4 units of glargine insulin from the current evening basal insulin dose if the weekly fasting glucose values were either consistently high or low, respectively. Using the usual dose for each week, each mealtime insulin dose was then corrected using 2-unit increments before each meal based on premeal SMBG values (simple correction scale) and meal size (larger or smaller than usual). In addition, subjects could add an insulin dose of 2 units for snacks exceeding one carbohydrate serving.

Subjects were asked to perform SMBG every day throughout the study before morning, midday, and evening meals, at bedtime, and when hypoglycemia was suspected based on symptoms. During the baseline period and before study visits at weeks 4, 12, 24, 36, and 44, subjects recorded in a diary 3 days of 7-point SMBG values (preprandial [3], 2-h postprandial [3], and bedtime measurements) along with insulin doses. They also recorded any adverse events, including hypoglycemic events, in the diary. Phone calls with subjects were conducted at weeks 1, 2, 3, 6, and 8 to assist them with self-titration.

### Outcomes

The primary endpoint was change in HbA1c from baseline to week 24, assessing for noninferiority of the patch to the pen. Prespecified secondary clinical endpoints included the percentage of subjects achieving HbA1c ≤7.0% (≤53 mmol/mol) at weeks 24 and 44; change in HbA1c from baseline to week 44; change in fasting plasma glucose from baseline to weeks 24 and 44; change in 3-day average 7-point SMBG values (mean daily blood glucose [MDBG]), including the coefficient of variation (CV) of MDBG, calculated from the 7-point SMBG profile over 3 days (21 values in total) at weeks 24 and 44; and change in 3-day average insulin doses (total daily dose, basal dose, mealtime dose) from baseline to weeks 24 and 44.

Safety measures included changes in body weight, clinically important changes in laboratory tests or vital signs, adverse events, device-related adverse events, and the frequency of hypoglycemic events (documented nonsevere symptomatic, documented nonsevere asymptomatic, and severe hypoglycemia). Documented nonsevere symptomatic hypoglycemia was defined as an event with typical symptoms of hypoglycemia accompanied by a measured glucose concentration by SMBG ≤70 mg/dL (≤3.9 mmol/L). Documented nonsevere asymptomatic hypoglycemia was defined as an event with measured glucose ≤70 mg/dL (≤3.9 mmol/L) without symptoms. Severe hypoglycemia was defined as an event requiring the assistance of another person to actively administer carbohydrate (including intravenous dextrose), glucagon, or other resuscitative actions.^23^ Nocturnal hypoglycemia was defined as any hypoglycemic event occurring between midnight and 6 A.M.

Self-reported outcomes evaluated were insulin regimen adherence at weeks 24 and 44^24,25^; subject-experience surveys at week 24 (ease of use, social interference), week 44 (diabetes management), and week 48 (preference for patch vs. pen) (developed at Calibra Medical); and changes in treatment satisfaction (Insulin Delivery System Rating Questionnaire)^13^ and quality of life (Diabetes Specific Quality of Life Scale)^26^ from baseline to week 24. A preference survey for health care providers regarding use of patch versus pen (developed by Calibra Medical) was used after the last subject completed the week 24 visit.

### Sample size

Sample size determination was based on the primary endpoint of HbA1c change from baseline to week 24. Assuming a true mean difference in change of HbA1c (patch vs. pen) of −0.1% (standard deviation [SD] 1.2%) (−1.1 [SD 13.1] mmol/mol), 250 completers (125 per arm) were required to achieve a power of 90% for noninferiority with a 0.4% margin. Allowing for a 20% discontinuation rate by week 24, the target number of randomized subjects was 312 (156 per arm).

### Statistical analyses

Unless otherwise noted, all tests of device effects were conducted at a two-sided alpha of 0.05, and two-sided confidence intervals (CIs) at 95%. For the primary endpoint, differences between treatment arms at week 24 were analyzed using ANCOVA with baseline HbA1c as covariate. Noninferiority (patch to pen) was concluded from the upper boundary of the two-sided 95% CI for change in HbA1c from baseline to week 24 being less than the inferiority margin of 0.4%.^27^ A modified intent-to-treat analysis set was used and included all the randomized intent-to-treat subjects who had a baseline HbA1c and at least one postbaseline HbA1c measurement (*n* = 274). If week 24 HbA1c measurement was missing, the last observation carried forward imputation method was used. Results are shown as mean ± standard error (SE), unless noted otherwise.

Secondary endpoints were tested for superiority, but results were to be interpreted inferentially only if noninferiority was demonstrated for the primary endpoint. Continuous endpoints were analyzed using the ANCOVA model described previously. The categorical endpoints were analyzed using a Cochran–Mantel–Haenszel test, with a type 1 error rate of 0.05. The change in HbA1c from weeks 24 to 44 was analyzed using a *t*-test for each treatment arm.

For analysis of the subject-experience surveys (weeks 24 and 44), a chi-squared test was used for comparisons of the two arms. For the participant-preference survey (week 48) and health care provider-experience survey (week 24), a within-group binomial test *P-*value was calculated after excluding neutral answers (Likert scale 3) and tested (Likert scale 4/5 vs. 1/2) with a null hypothesis probability (Likert scale 4/5) of 0.5; the hypothesis tested is that there are more favorable ratings than unfavorable ratings. A chi-squared test was used for the comparisons of the participant-preference survey (week 48) in the two crossover groups (patch use for 44 weeks vs. 4 weeks).

## Results

### Subjects

Baseline characteristics are shown in Table 1. Subjects (*n* = 278) were enrolled between July 2015 and August 2016 and were randomized 1:1 to either patch (*n* = 139) or pen (*n* = 139). Of those, 241 (87%) and 216 (78%) completed week 24 and week 44 assessments, respectively. The study population was 60% male, with an average age of 59.2 ± 8.9 years, mean duration of diabetes 15.0 ± 7.5 years, and BMI 32.6 ± 4.4 kg/m^2^. The ethnic distribution of subjects was 89% Caucasian, 8% African American, 2% Asian, 1% American Indian, and 1% other. Geographic representation was 78% from the United States, 12% from France, 8% from the United Kingdom, and 2% from Germany.

**Table 1.** Baseline Characteristics and Changes in Efficacy Endpoints from Baseline to Weeks 24 and 44

	*Week 24 (mITT)*			*Week 44 (mITT)*		
	*Patch*	*Pen*	*Patch vs. pen*	*Patch*	*Pen*	*Patch vs. pen*
HbA1c	*n* = 136	*n* = 138		*n* = 108	*n* = 109	
Baseline, mean ± SE, %	8.6 ± 0.1	8.7 ± 0.1		8.6 ± 0.1	8.6 ± 0.1	
Week 24 or 44, mean ± SE, %	7.0 ± 0.1	7.1 ± 0.1		7.0 ± 0.1	7.0 ± 0.1	
LS mean change ± SE, %	−1.7 ± 0.1	−1.6 ± 0.1	−0.1 ± 0.1	−1.6 ± 0.1	−1.6 ± 0.1	0.0 ± 0.1
95% CI	−1.86 to −1.53	−1.77 to −1.44	−0.32 to 0.14	−1.83 to −1.43	−1.83 to −1.43	−0.28 to 0.28
*P*			0.45			0.99
Baseline, mean ± SE, mmol/mol	70.8 ± 0.9	71.8 ± 0.9		70.1 ± 1.0	70.5 ± 1.0	
Week 24 or 44, mean ± SE, mmol/mol	52.7 ± 0.9	54.0 ± 1.0		52.5 ± 1.2	52.6 ± 1.1	
LS mean change ± SE, mmol/mol	−18.5 ± 0.9	−17.5 ± 0.9	−1.0 ± 1.3	−17.8 ± 1.1	−17.8 ± 1.1	0.02 ± 1.57
95% CI	−20.3 to −16.7	−19.3 to −15.7	−3.5 to 1.6	−20.0 to −15.6	−20.0 to −15.6	−3.1 to 3.1
*P*			0.45			0.99
Fasting plasma glucose	*n* = 135	*n* = 137		*n* = 106	*n* = 109	
Baseline, mean ± SE, mg/dL	161.5 ± 4.9	169.0 ± 5.3		154.5 ± 5.6	161.9 ± 5.1	
Week 24 or 44, mean ± SE, mg/dL	136.1 ± 4.6	138.8 ± 4.3		138.3 ± 4.7	138.5 ± 4.6	
LS mean change ± SE, mg/dL	−28.3 ± 4.3	−27.4 ± 4.2	−0.9 ± 6.0	−19.1 ± 4.5	−20.6 ± 4.4	1.5 ± 6.3
95% CI	−36.7 to −19.9	−35.7 to −19.0	−12.8 to 10.9	−28.0 to −10.2	−29.3 to −11.8	−11.0 to 14.0
*P*			0.88			0.81
MDBG	*n* = 100	*n* = 110		*n* = 89	*n* = 98	
Baseline, mean ± SE, mg/dL	199.3 ± 4.1	202.5 ± 5.0		198.6 ± 4.1	200.6 ± 5.1	
Week 24 or 44, mean ± SE, mg/dL	140.6 ± 2.6	146.6 ± 3.0		143.4 ± 3.9	142.2 ± 2.6	
LS mean change ± SE, mg/dL	−60.1 ± 2.8	−54.7 ± 2.6	−5.4 ± 3.8	−56.1 ± 3.2	−57.6 ± 3.1	1.5 ± 4.5
95% CI	−65.5 to −54.6	−59.9 to −49.5	−12.9 to 2.2	−62.4 to −49.7	−63.7 to −51.5	−7.3 to 10.3
*P*			0.16			0.73
CV of MDBG^a^	*n* = 100	*n* = 109		*n* = 89	*n* = 97	
Baseline, mean ± SE, %	10.3 ± 0.5	10.5 ± 0.6		10.5 ± 0.6	10.7 ± 0.6	
Week 24 or 44, mean ± SE, %	11.0 ± 0.7	11.8 ± 0.7		9.4 ± 0.7	12.0 ± 0.8	
LS mean change ± SE, %	0.6 ± 0.7	1.4 ± 0.7	−0.7 ± 1.0	−1.2 ± 0.8	1.4 ± 0.8	−2.6 ± 1.1
95% CI	−0.8 to 2.1	0.0 to 2.7	−2.7 to 1.2	−2.8 to 0.4	−0.4 to 3.0	−4.8 to −0.4
*P*			0.46			0.02
Total daily insulin dose	*n* = 98	*n* = 106		*n* = 88	*n* = 94	
Baseline, mean ± SE, units/day	43.4 ± 1.9	49.5 ± 2.2		42.6 ± 2.0	49.0 ± 2.4	
Week 24 or 44, mean ± SE, units/day	114.2 ± 5.6	133.0 ± 5.8	−10.8 ± 7.2	130.9 ± 8.2	140.5 ± 8.2	
LS mean change ± SE, units/day	71.8 ± 5.2	82.6 ± 5.0	−25.1, 3.5	89.2 ± 7.9	90.7 ± 7.6	−1.54 ± 11.1
95% CI	61.5 to 82.0	72.7 to 92.4		73.6 to 104.8	75.6 to 105.8	−23.4 to 20.3
*P*			0.14			0.89
Total daily insulin dose	*n* = 98	*n* = 106		*n* = 88	*n* = 94	
Baseline, mean ± SE, units/kg	0.46 ± 0.02^b^	0.52 ± 0.02^b^		0.46 ± 0.02	0.51 ± 0.03	
Week 24 or 44, mean ± SE, units/kg	1.17 ± 0.05	1.32 ± 0.06		1.33 ± 0.08	1.38 ± 0.08	
LS mean change ± SE, units/kg	0.71 ± 0.05	0.80 ± 0.05	−0.09 ± 0.07	0.87 ± 0.08	0.86 ± 0.08	0.01 ± 0.11
95% CI	0.61 to 0.81	0.70 to 0.90	−0.24 to 0.05	0.71 to 1.03	0.71 to 1.02	−0.21 to 0.23
*P*			0.20			0.93
Basal insulin dose	*n* = 97	*n* = 106		*n* = 88	*n* = 93	
Baseline, mean ± SE, units/kg	0.24 ± 0.01	0.27 ± 0.01		0.24 ± 0.01	0.28 ± 0.02	
Week 24 or 44, mean ± SE, units/kg	0.48 ± 0.02	0.53 ± 0.02		0.52 ± 0.03	0.55 ± 0.03	
LS mean change ± SE, units/kg	0.24 ± 0.02	0.26 ± 0.02	−0.02 ± 0.03	0.27 ± 0.03	0.28 ± 0.03	−0.002 ± 0.04
95% CI	0.20 to 0.28	0.22 to 0.30	−0.08 to 0.03	0.22 to 0.33	0.22 to 0.33	−0.08 to 0.07
*P*			0.38			0.96
Bolus insulin dose	*n* = 97	*n* = 106		*n* = 88	*n* = 94	
Baseline, mean ± SE, units/kg	0.22 ± 0.01	0.25 ± 0.01		0.22 ± 0.01	0.24 ± 0.01	
Week 24 or 44, mean ± SE, units/kg	0.70 ± 0.04	0.79 ± 0.04		0.810 ± 0.06	0.84 ± 0.06	
LS mean change ± SE, units/kg	0.48 ± 0.04	0.54 ± 0.04	−0.06 ± 0.05	0.60 ± 0.06	0.60 ± 0.06	0.001 ± 0.08
95% CI	0.41 to 0.55	0.47 to 0.61	−0.16 to 0.04	0.48 to 0.71	0.478 to 0.71	−0.16 to 0.16
*P*			0.22			0.99
Insulin ratio—basal:bolus	*n* = 96	*n* = 106		*n* = 88	*n* = 93	
Baseline, mean ± SE, %	1.12 ± 0.03^b^	1.14 ± 0.03^b^		1.12 ± 0.03	1.19 ± 0.04	
Week 24 or 44, mean ± SE, %	0.75 ± 0.03	0.79 ± 0.04		0.79 ± 0.05	0.81 ± 0.04	
LS mean change ± SE, %	−0.38 ± 0.04	−0.35 ± 0.03	−0.03 ± 0.05	−0.36 ± 0.05	−0.35 ± 0.04	−0.01 ± 0.06
95% CI	−0.45 to −0.31	−0.41 to −0.28	−0.13 to 0.06	−0.44 to −0.27	−0.44 to −0.26	−0.13 to 0.12
*P*			0.49			0.93
Body weight	*n* = 133	*n* = 128		*n* = 114	*n* = 116	
Baseline, mean ± SE, kg	92.5 ± 1.4	96.1 ± 1.5		92.6 ± 1.6	96.7 ± 1.6	
Week 24 or 44, mean ± SE, kg	96.3 ± 1.5	100.1 ± 1.6		97.6 ± 1.8	102.1 ± 1.8	
LS mean change ± SE, kg	3.9 ± 0.4	4.0 ± 0.4	−0.07 ± 0.57	5.1 ± 0.5	5.3 ± 0.5	−0.2 ± 0.7
95% CI	3.1 to 4.7	3.2 to 4.8	−1.18 to 1.04	4.1 to 6.0	4.3 to 6.2	−1.6 to 1.2
*P*			0.91			0.79
Hypoglycemic episodes,^c^*n* (%), ITT population (*n* = 278)						
Any^d^	116 (83.5)	120 (86.3)	*P* = 0.50	121 (87.1)	125 (89.9)	*P* = 0.45
Symptomatic	105 (75.5)	107 (77.0)	*P* = 0.78	113 (81.3)	115 (82.7)	*P* = 0.75
Asymptomatic	77 (55.4)	76 (54.7)	*P* = 0.90	94 (67.6)	90 (64.7)	*P* = 0.61
Severe^e^	1 (0.7)	2 (1.4)	*P* = 0.56	3 (2.2)	3 (2.2)	*P* = 1.00
Nocturnal^f^	50 (36.0)	52 (37.4)	*P* = 0.80	60 (43.2)	74 (53.2)	*P* = 0.09

^a^ CV was defined as the measure of glycemic variability relative to the mean (SD/mean), calculated from 7-point SMBG over 3 days.

^b^ Baseline is defined as the Visit 3 (week 0) measurement.

^c^ Defined as plasma glucose ≤70 mg/dL (≤3.9 mmol/L) accompanied by typical symptoms of hypoglycemia.

^d^ Some patients experienced multiple episodes.

^e^ Defined as requiring third-party assistance.

^f^ Any hypoglycemic event occurring between the hours of 12 midnight and 6 A.M.

CI, confidence interval; CV, coefficient of variation; HbA1c, glycated hemoglobin; ITT, intent to treat; LS, least squares; MDBG, mean daily blood glucose; mITT, modified ITT; SD, standard deviation; SE, standard error; SMBG, self-monitoring of blood glucose.

### Efficacy outcomes

#### Primary endpoint

**FIG. 2.**
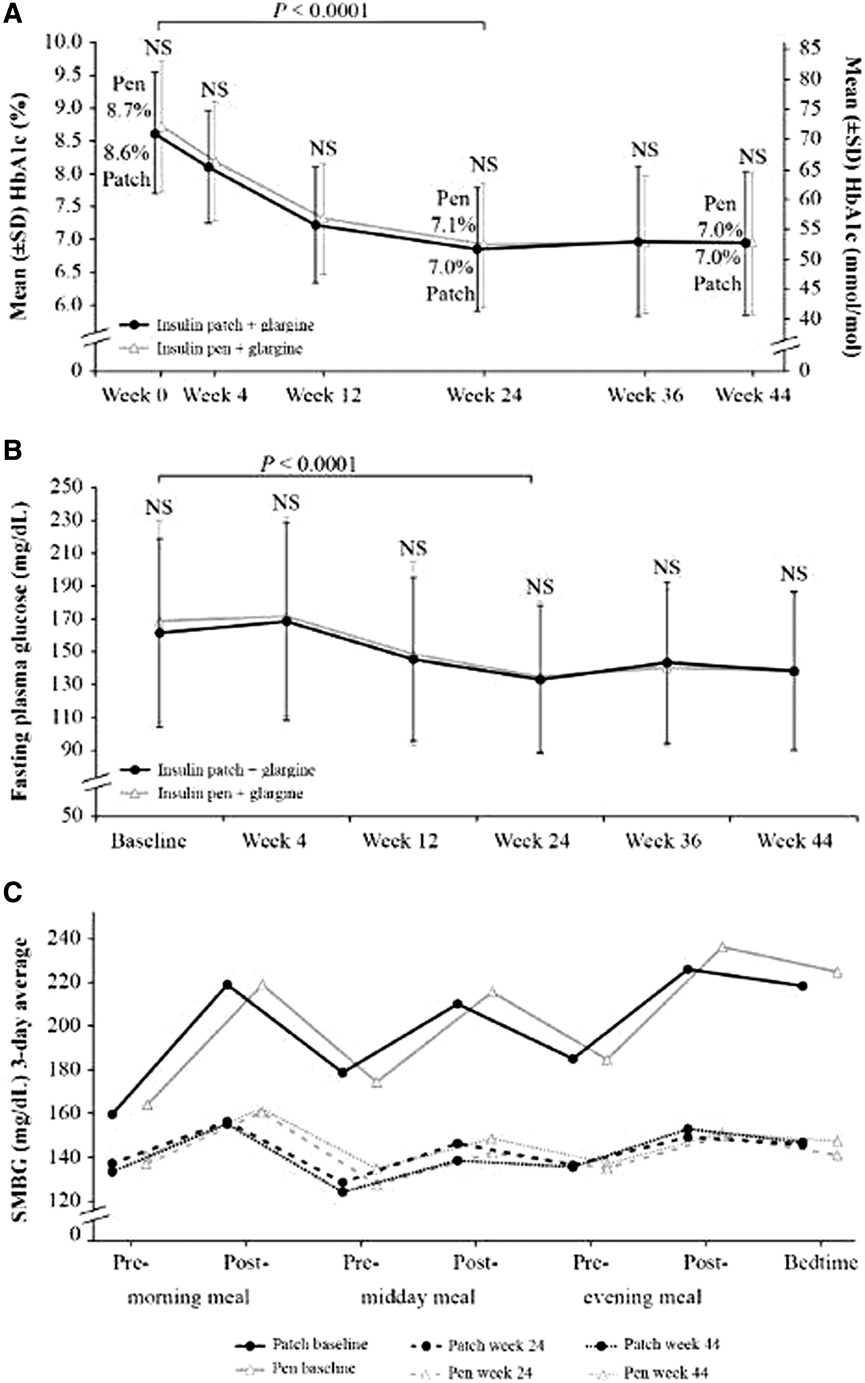
Results for glycemic control. **(A)** HbA1c from baseline to week 44 in patch versus pen users. **(B)** Fasting plasma glucose from baseline to week 44 in patch versus pen users. **(C)** Seven-point SMBG profile at baseline, week 24, and week 44 in patch versus pen users. A significant reduction in SMBG was observed from baseline to weeks 24 and 44 for each of the seven time points (*P* < 0.0001) in both treatment arms. HbA1c, glycated hemoglobin; NS, nonsignificant between treatment arms; SMBG, self-monitoring of blood glucose.

The least squares (LS) mean change in HbA1c from baseline to week 24 (± SE) was significant for both arms (−1.7% ± 0.1% [−18.5 ± 0.9 mmol/mol] and −1.6% ± 0.1% [−17.5 ± 0.9 mmol/mol] for patch and pen, respectively; *P* < 0.0001) (Table 1 and Fig. 2A); the treatment arm comparison met the predefined threshold for noninferiority of patch versus pen (*P* < 0.0001). Improvement in glycemic control was maintained from baseline to week 44 (LS mean change −1.6% ± 0.1% [−17.8 ± 1.1 mmol/mol] and −1.6% ± 0.1% [−17.8 ± 1.1 mmol/mol] for patch and pen, respectively).

#### Secondary endpoints

A total of 63% of patch users and 56% of pen users achieved HbA1c ≤7.0% (≤53 mmol/mol) at week 24 (odds ratio [OR] 1.3, SE 0.25, 95% CI 0.81–2.14; *P* = 0.26). The proportions of patch and pen users who achieved HbA1c ≤7.0% (≤53 mmol/mol) at week 44 rose to 65% and 63%, respectively (OR 1.2, SE 0.28, 95% CI 0.64–1.93; *P* = 0.71). The LS mean change (± SE) in fasting plasma glucose was significant for both patch and pen from baseline to week 24 (*P* < 0.0001) (Table 1 and Fig. 2B). The fasting glucose target was 71–130 mg/dL and the 24-week fasting glucose was 136.1 ± 4.6 and 138.8 + 4.3 mg/dL in the patch and pen groups, respectively, indicating that there may still have been room for additional basal insulin adjustment. A significant decrease in the 7-point SMBG was observed at weeks 24 and 44 for both patch and pen (*P* < 0.0001) (Table 1 and Fig. 2C). The CV of the MDBG of 7-point SMBG decreased from baseline to week 44 for patch (−1.2% ± 0.8%); in contrast, an increase in 3-day CV of 7-point SMBG was observed in the pen treatment arm (1.4% ± 0.8%). The difference in change between patch and pen was statistically significant (−2.6% ± 1.1%; 95% CI −4.8 to −0.4; *P* = 0.022) (Table 1). Basal and bolus insulin doses, insulin total daily dose, units/day, and units/kg increased from baseline to weeks 24 and 44 in both treatment arms, with no significant difference between the groups (Table 1). Basal:bolus insulin ratio decreased from 1.1 to 0.8 from baseline to weeks 24 and 44 similarly in both groups (Table 1).

### Safety outcomes

Average body weight increased similarly in the two treatment arms by week 24 (patch: 3.9 ± 0.4 kg; pen: 4.0 ± 0.4 kg) and week 44 (patch: 5.1 ± 0.5 kg; pen: 5.3 ± 0.5 kg), with no significant difference between groups (Table 1). Similar percentages of subjects in both treatment arms experienced hypoglycemic events (Table 1). Three episodes of severe hypoglycemia per treatment arm were reported (2.2% per arm) over the 44 weeks (Table 1), three of which were considered serious adverse events (one in a subject using the patch and two in subjects using the pen) and related to intensifying insulin therapy. Adverse events at week 44 were similar between treatment arms (71.9% and 71.2% for patch vs. pen, respectively). Serious adverse events were reported in 7.2% and 9.4% of subjects using patch and pen, respectively; most of these (90% and 85%, respectively) were deemed unrelated to intensifying insulin therapy. Three deaths occurred—one subject using patch and two subjects using the pen; these were unrelated to intensifying insulin therapy. Five subjects discontinued the study because of serious adverse events other than death: three subjects who were using the patch (angina, coronary artery disease, severe hypoglycemia) and two who were using the pen (bone abscess, severe hypoglycemia).

At week 48, 9% of subjects (25/278) using the patch reported 33 device-related adverse effects, including bleeding at the insertion site (7 events); injection-site bruising and contusion (5 events); insertion-site pain (4 events); injection-site mass, edema, and vesicles (4 events); insertion-site infection (2 events); discomfort under the tape (injection-site pruritus, application-site pruritus, injection-site erythema, injection-site irritation, injection-site rash, miliaria, and dermatitis) (10 events); and excess sweating under the tape (1 event). None of the device-related adverse effects was severe; in fact, the majority were mild in severity. No serious adverse device-related effects were reported during the study. Two subjects permanently discontinued the patch during the study due to adverse device effects of moderate severity (one injection-site pruritus and one injection-site pain).

The technical performance of the patch device was robust. Overall, 102 device complaints were reported by 52 patients (21.1%) through week 44. The most common device complaints included discomfort related to the insertion site (30 events), button safety (31 events), and product usability (11 events).

### Subject-reported outcomes

Good adherence to mealtime and snack insulin regimens within the prior 30 days was reported by 79% ± 18% and 78% ± 16% of subjects using the patch and pen, respectively, at week 24 (*P* = 0.68), and 81% ± 15% and 81% ± 17% of subjects at week 44, respectively (*P* = 0.78), with no significant differences between treatment arms.

Subject-reported outcomes are shown in Table 2. Changes in treatment satisfaction with their insulin-delivery system from baseline to week 24 favored patch over pen for all measures; comparisons for overall satisfaction and ease-of-use scores were significant. Changes in quality of life from baseline to week 24 favored the patch for 6/7 measures; comparisons of daily functions and diet restrictions were significant. The scores from the subject-experience survey at week 24 were positive on all measures for both patch and pen; participants using the patch recorded significantly higher ratings compared with those using the pen for 7 of 11 measures. At week 44, four additional questions about perspectives on diabetes management were asked and both groups showed similarly positive responses.

**Table 2.** Subject-Reported Outcomes

	*Patch, LS mean ± SE*	*Pen, LS mean ± SE*	*Patch vs. pen, LS mean ± SE*^a^	P*-value (patch vs. pen)*
Change in insulin-delivery-system treatment satisfaction, baseline to week 24				
	*n* = 124	*n* = 117		
Overall satisfaction^b^	−0.7 ± 0.1	−0.5 ± 0.1	−0.3 ± 0.1	<0.01
Satisfaction with ease of use^c^	13.6 ± 2.0	4.5 ± 2.0	9.2 ± 2.8	<0.01
Interference with daily activities^b^	2.5 ± 2.2	3.3 ± 2.2	−0.8 ± 3.1	0.79
Helping with glucose control^c^	16.4 ± 2.0	13.2 ± 2.0	3.1 ± 2.9	0.27
Worry about glucose control^b^	−3.3 ± 1.6	−0.5 ± 1.6	−2.8 ± 2.3	0.22
Feelings about yourself and your life^c^	3.8 ± 1.1	2.5 ± 1.1	1.3 ± 1.6	0.41
Change in diabetes-specific quality of life, baseline to week 24				
	*n* = 124	*n* = 123		
Daily functions^c^	2.4 ± 1.4	−2.0 ± 1.4	4.3 (2.0)	0.03
Diet restrictions^c^	6.2 ± 1.3	1.9 ± 1.3	4.2 (1.8)	0.02
Treatment goals^c^	0.9 ± 1.0	−0.3 ± 1.0	1.1 (1.5)	0.44
Treatment satisfaction^b^	−13.2 ± 1.4	−11.3 ± 1.4	−1.9 (2.0)	0.33
Physical complaints^c^	4.5 ± 1.3	2.5 ± 1.3	2.0 (1.8)	0.26
Emotional burdens^c^	6.0 ± 1.4	3.0 ± 1.4	3.0 (1.9)	0.12
Social problems^c^	2.5 ± 1.1	0.6 ± 1.1	1.9 (1.6)	0.22

	*Patch, % (95% CI)*	*Pen, % (95% CI)*	*Odds ratio (95% CI)*	P*-value (patch vs. pen)*
Subject-experience survey at week 24, % favorable^c,d^				
	*n* = 123	*n* = 109		
Dosed without attracting attention	93.5 (89.8–97.2)	68.8 (61.5–76.1)	6.5 (2.9–14.8)	<0.0001
Taking mealtime insulin was painless	90.2 (85.8–94.6)	70.4 (63.1–77.6)	3.9 (1.9–8.0)	<0.001
Could do things on the spur of the moment	87.0 (82.0–92.0)	66.7 (59.2–74.1)	3.3 (1.7–6.5)	<0.001
Always had mealtime insulin with me	91.2 (86.8–95.5)	72.5 (65.3–79.8)	3.9 (1.8–8.5)	<0.001
Felt comfortable using it socially	89.3 (84.7–93.9)	71.0 (63.8–78.2)	3.4 (1.7–7.0)	<0.001
Taking mealtime insulin was easy	92.7 (88.8–96.5)	78.0 (71.5–84.5)	3.6 (1.6–8.1)	<0.01
Recommend for mealtime insulin	91.0 (86.7–95.2)	78.9 (72.5–85.3)	2.7 (1.2–5.8)	0.01
Better relationship with my health care provider	74.6 (68.1–81.1)	66.1 (58.6–73.5)	1.5 (0.9–2.7)	0.16
Confident that dosed correctly	91.9 (87.8–95.9)	89.9 (85.2–94.7)	1.3 (0.5–3.1)	0.60
Engaged with managing my diabetes	90.1 (85.6–94.6)	88.9 (83.9–93.9)	1.1 (0.5–2.6)	0.77
Made improvements to my diabetes	87.0 (82.0–92.0)	86.2 (80.8–91.7)	1.1 (0.5–2.3)	0.87
Subject-experience survey at week 44, % favorable^c,d^				
	*n* = 113	*n* = 108		
I feel confident with managing my insulin	93.8 (90.1–97.5)	94.4 (90.8–98.1)	0.9 (0.3–2.7)	0.84
I have peace of mind in managing my diabetes for my future	89.4 (84.6–94.1)	91.7 (87.3–96.0)	0.8 (0.3–1.9)	0.56
Has allowed me to fit diabetes management into my life	92.9 (89.0–96.9)	89.8 (85.0–94.6)	1.5 (0.6–3.9)	0.41
I am taking positive steps with my diabetes management	88.5 (83.6–93.4)	92.6 (88.4–96.7)	0.6 (0.2–1.5)	0.30

	*Used patch for 44 weeks, % (95% CI)* P	*Used patch for 4 weeks, % (95% CI)* P	*Odds ratio (95% CI)*	P*-value (44 weeks vs. 4 weeks)*
Subject-preference survey at week 48, % prefer^c,d^				
	*n* = 108	*n* = 106		
I am more satisfied using the patch compared with the pen for mealtime insulin therapy	75.0 (68.1–81.9) <0.0001	63.2 (55.5–70.9) <0.0001	1.4 (0.69–3.01)	0.34
I prefer using the patch compared with the pen for mealtime insulin therapy	72.2 (65.1–79.3) <0.0001	57.5 (49.7–65.4) <0.0001	1.5 (0.77–3.10)	0.22
With the patch compared with the pen, I had to carry fewer diabetes supplies with me	82.2 (76.2–88.3) <0.0001	82.1 (75.9–88.2) <0.0001	1.7 (0.65–4.61)	0.27
With the patch compared with the pen, I feel less constrained with my diabetes management	75.9 (69.2–82.7) <0.0001	66.0 (58.5–73.6) <0.0001	1.3 (0.53–3.08)	0.58
With the patch compared with the pen, I feel more freedom with my diabetes management	77.8 (71.2–84.4) <0.0001	60.0 (52.6–68.2) <0.0001	1.6 (0.73–3.60)	0.24
I would recommend the patch compared with the pen to other patients who are on mealtime insulin therapy	71.3 (64.1–78.5) <0.0001	65.1 (57.5–72.7) <0.0001	1.4 (0.60–3.34)	0.42
I want to switch from the pen to the patch	69.4 (62.2–76.7) <0.0001	50.9 (43.0–58.9) 0.02	2.2 (1.15–4.30)	0.02

^a^ LS mean that is greater than 2 SE represents a statistically significant (*P* < 0.05) change.

^b^ Lower score is better.

^c^ Higher score is better.

^d^ ”Favorable” and “Prefer” are defined as 4 or 5 on a Likert scale of 1–5, with 1 = strongly disagree, 2 = disagree, 3 = neutral, 4 = agree, 5 = strongly agree.

The subject-preference survey at week 48 showed that significantly more subjects preferred the patch to pen for all measures in those who used the patch for 44 or 4 weeks after crossover. Most participants stated that they would like to switch from pen to patch. Longer patch use (44 weeks) was associated with a significantly higher percentage of subjects (*P* = 0.02) wanting to switch from pen to patch when compared with subjects using the patch for 4 weeks.

### Health care provider experience

Health care providers (*N* = 89) gave significant, favorable ratings for the patch for all measures after 24 weeks (Table 3). Most health care providers (91.1%, 95% CI 84.8–97.3; *P* < 0.0001) preferred the patch to pen to advance subjects with type 2 diabetes from basal to basal–bolus insulin. In addition, 89% of health care providers reported that it took less than 30 min to train subjects on the use of the patch.

**Table 3.** Health Care Provider-Experience Survey (N = 89)

	*Response, % favorable (95% CI)*^a,b^	P
I am satisfied with the patch	85.4 (79.2–91.6)	<0.0001
I would prescribe the patch to patients who need bolus insulin	84.1 (77.7–90.5)	<0.0001
The patch will help patients overcome barriers to insulin injections (syringe/pen)	80.9 (74.0–87.8)	<0.0001
Easy for type 2 diabetes patients using the patch to advance from basal to basal–bolus insulin	79.8 (72.8–86.8)	<0.0001
I would prescribe the patch to MDI patients not at goal	78.4 (71.2–85.6)	<0.0001
Training patients to use the patch was easy	74.2 (66.5–81.8)	<0.0001
I would initiate type 2 diabetes patients uncontrolled on basal to basal–bolus with the patch	73.9 (66.2–81.6)	<0.0001
I observed positive diabetes management behavior changes with patients using the patch	70.8 (62.9–78.7)	<0.0001
When patients used the patch, they became engaged with their diabetes management	68.5 (60.4–76.6)	<0.0001
I would prescribe the patch for MDI patients at goal	67.0 (58.8–75.3)	<0.0001
I prefer the patch to pen to advance type 2 diabetes patients from basal to basal–bolus insulin	91.1 (84.8–97.3)^c^	<0.0001
The patch will help me transition patients to basal–bolus therapy sooner/faster	87.5 (79.6–95.4)^c^	<0.0001
With the patch, I had a more gratifying relationship with my patients	73.8 (62.7–85.0)^c^	<0.01

^a^ ”Favorable” is defined as 4 or 5 on a Likert scale of 1–5 (1 = strongly disagree, 2 = disagree, 3 = neutral, 4 = agree, 5 = strongly agree).

^b^ Higher score is better.

^c^ Health care provider who expressed a preference excluding neutral responses of >20%.

MDI, multiple daily injections.

## Discussion

This study of subjects who previously had not achieved glycemic goals despite taking moderately high doses of basal insulin (∼0.5 units/kg) with or without other antihyperglycemic agents showed a substantial and clinically significant decrease in HbA1c 24 weeks after initiation of mealtime insulin therapy. This decrease persisted at 44 weeks. A large percentage (83%) of the decrease in HbA1c was observed after just 12 weeks of therapy. Within 24 weeks, more than half the subjects in this study achieved the HbA1c goal of ≤7.0% (≤53 mmol/mol), increasing to about two-thirds of subjects by week 44. There were no significant differences between treatment arms in measures of HbA1c or overall mean glucose levels. Subjects in both treatment arms gained similar amounts of weight. In the study, any device-related adverse effects were localized to the insertion site and the majority were considered mild in severity. As the patch can be worn up to 3 days, the possibility for insertion-site and adhesion complaints might be expected,^28^ but was quite low. Overall, the patch demonstrated a good safety profile, with no serious adverse device-related events and a similarly low percentage of study-related adverse events with the patch versus pen (0.7% vs. 1.5%, respectively).

In this study, systematic insulin intensification using a simplified dosing algorithm for the subject alongside health care provider oversight and engagement resulted in high adherence and good glycemic control in both groups. After nearly a 1-year follow-up, about two-thirds of the subjects had reached and maintained the treatment target of HbA1c ≤7.0% (≤53 mmol/mol). In previous reports, only 30% of patients with diabetes in the United States on insulin therapy achieved an HbA1c of ≤7.0% (≤53 mmol/mol).^4,5^ Moreover, in our study, there was a statistically significant, although marginal, reduction in glycemic variability (assessed by measuring the CV of SMBG measurements on multiple, 7-point SMBG profiles over 3 days) with the patch versus pen after 44 weeks. The reduction in glycemic variability was not apparent at week 24. These findings are in alignment with the results of a smaller feasibility study showing that the patch reduced glycemic variability compared with mealtime insulin by injection.^28^ This is despite subjects in both groups reporting similar adherence and achieving similar daily mean glucose levels. One hypothesis is that the patch made it easier to maintain consistent glucose levels throughout the day, rather than subjects needing to make larger corrective doses to stabilize glucose levels as the day progressed. For example, at week 24, there was a greater reduction for patch versus pen in mean glucose premidday meal (*P* = 0.01) and postmidday meal (*P* = 0.05). The reduction is possibly due to greater adherence to either the breakfast or the lunch mealtime bolus, however, this is a post hoc interpretation that should be regarded with caution. Nevertheless, lower glucose variability is associated with increased quality of life^29^ and decreased risk of pathophysiologic changes associated with vascular complications.^30–32^

Self-reported adherence to basal–bolus insulin therapy was high in this study, potentially contributing to the observed improvements in glycemic control and quality of life. Insulin therapy generally is associated with lower user satisfaction and quality of life^33^; however, in this study, advancing to mealtime insulin therapy led to improvement in several subject- and provider-reported outcomes. Overall, the patch was associated with greater device satisfaction, a more positive experience, and preference for initiating mealtime insulin when compared with the pen. The quality-of-life improvements in this study associated with the patch suggest that this device can address many issues that affect adherence to mealtime insulin in real-world therapy implementation, including interference with lifestyle, daily activities, travel, social situations, embarrassment, and injection pain.^16,17^ In addition, these findings confirm the results of a smaller feasibility study showing that the patch offered better quality of life and higher device satisfaction compared with mealtime insulin by injection while providing similar glycemic control and safety.^28^

Most health care providers reported that the patch was easy to use, required a short time for subject training, and was preferred over a pen for initiating mealtime insulin. These factors, combined with health care provider recognition of the positive experience of subjects using the patch, might reduce barriers to providers recommending and implementing mealtime insulin therapy in real-world settings. If clinical inertia can be reduced, patients may benefit from reduced exposure to hyperglycemia, which is likely to reduce the risk of diabetes complications.^34^

The strengths of this study include participation by 62 centers from 4 countries, a randomized design with a comparator arm, treatment crossover to permit direct comparison of subject preference, a formal protocol for insulin adjustment/titration, long duration (48 weeks), and a high rate of retention (78% of subjects completed week 44 assessments). A limitation was that it was not possible to blind subjects or health care providers to the device used, leading to potential expectation effects. This is a common limitation in studies for medical devices.

## Conclusions

This study demonstrated clinically significant improvements in glycemic control after the addition of mealtime insulin to a basal insulin regimen using either the patch or an insulin pen. The simplified dosing algorithm was safe and effective, enabling two-thirds of subjects to reach an HbA1c ≤7.0% (≤53 mmol/mol). Similar increases in body weight and low numbers of severe hypoglycemic events were observed in the two treatment arms. Glycemic variability was marginally reduced for subjects using the patch compared with the pen. Overall, subjects and health care providers preferred the patch over the pen for implementing basal–bolus insulin therapy. The patch can contribute to safely achieving glycemic control for people initiating basal–bolus insulin therapy. It can potentially reduce patient and provider resistance to initiating mealtime insulin therapy and improve patient adherence and persistence, resulting in improved glycemic control over time.

## Statements of Assistance

Statistical analysis and clinical operations support was provided by Shawna Brown, Jamal Gasmi, Nickolas Halbert, Jennifer Harness, Roland Hausknecht, Brenda Jorissen, Juan Liang, Celeste Morris, Andrew Nagel, Krisztina Nagy, Maria Nikiforou, Amanda Reuss, Tracie Ruther, Beth Smith, Steve Strassell, and Gretchen Williamson, of Medpace, funded by Calibra Medical.

Editorial support in the preparation of this article was provided by Martina Fuchsberger, PhD, of Excerpta Medica, funded by Calibra Medical.

## References

[1] World Health Organisation: Global Report on Diabetes. 2016 www.who.int/diabetes/global-report/en (accessed 81, 2018)

[2] Hameed I, Masoodi SR, Mir SA, et al.: Type 2 diabetes mellitus: from a metabolic disorder to an inflammatory condition. World J Diabetes 2015;6:598–612 25987957 10.4239/wjd.v6.i4.598 PMC4434080

[3] American Diabetes Association: Glycemic targets: standards of medical care in diabetes—2019. Diabetes Care 2019;42(Suppl 1):S61–S70 30559232 10.2337/dc19-S006

[4] Stark-Casagrande S, Fradkin JE, Saydah SH, et al.: The prevalence of meeting A1C, blood pressure, and LDL goals among people with diabetes, 1988–2010. Diabetes Care 2013;36:2271–2279 23418368 10.2337/dc12-2258 PMC3714503

[5] Selvin E, Parrinello CM, Daya N, Bergenstal RM: Trends in insulin use and diabetes control in the U.S.: 1988–1994 and 1999–2012. Diabetes Care 2016;39:e33–e35 26721815 10.2337/dc15-2229 PMC4764038

[6] Inzucchi SE, Bergenstal RM, Buse JB, et al.: Management of hyperglycaemia in type 2 diabetes, 2015: a patient-centred approach. Update to a position statement of the American Diabetes Association and the European Association for the Study of Diabetes. Diabetes Care 2015;38:140–149 25538310 10.2337/dc14-2441

[7] Aschner P: New IDF clinical practice recommendations for managing type 2 diabetes in primary care. Diabetes Res Clin Pract 2017;132:169–170 28962686 10.1016/j.diabres.2017.09.002

[8] National Institute for Health and Care Excellence: Type 2 Diabetes in Adults: Management. (NG 28). Published December 2015; updated May 2017 https://www.nice.org.uk/guidance/ng28 (accessed 114, 2018)

[9] Owens DR: Clinical evidence for the earlier initiation of insulin therapy in type 2 diabetes. Diabetes Technol Ther 2013;15:776–785 23786228 10.1089/dia.2013.0081 PMC3757533

[10] Khunti K, Wolden ML, Thorsted BL, et al.: Clinical inertia in people with type 2 diabetes: a retrospective cohort study of more than 80,000 people. Diabetes Care 2013;36:3411–3417 23877982 10.2337/dc13-0331 PMC3816889

[11] Khunti K, Nikolajsen A, Thorsted BL, et al.: Clinical inertia with regard to intensifying therapy in people with type 2 diabetes treated with basal insulin. Diabetes Obes Metab 2016;18:401–409 26743666 10.1111/dom.12626 PMC5067688

[12] Khunti K, Millar-Jones D: Clinical inertia to insulin initiation and intensification in the UK: a focused literature review. Prim Care Diabetes 2017;11:3–12 27727005 10.1016/j.pcd.2016.09.003

[13] Peyrot M, Rubin RR: Validity and reliability of an instrument for assessing health-related quality of life and treatment preferences: the Insulin Delivery System Rating Questionnaire. Diabetes Care 2005;28:53–58 15616233 10.2337/diacare.28.1.53

[14] Hayes RP, Fitzgerald JT, Jacober SJ: Primary care physician beliefs about insulin initiation in patients with type 2 diabetes. Int J Clin Pract 2008;62:860–868 18393965 10.1111/j.1742-1241.2008.01742.x PMC2408662

[15] Sorli C, Heile MK: Identifying and meeting the challenges of insulin therapy in type 2 diabetes. J Multidiscip Healthc 2014;7:267–282 25061317 10.2147/JMDH.S64084 PMC4086769

[16] Peyrot M, Barnett AH, Meneghini LF, Schumm-Draeger PM: Factors associated with injection omission/non-adherence in the Global Attitudes of Patients and Physicians in Insulin Therapy Study. Diabetes Obes Metab 2012;14:1081–1087 22726104 10.1111/j.1463-1326.2012.01636.x

[17] Peyrot M, Barnett AH, Meneghini LF, Schumm-Draeger PM: Insulin adherence behaviours and barriers in the multinational Global Attitudes of Patients and Physicians in Insulin Therapy study. Diabet Med 2012;29:682–689 22313123 10.1111/j.1464-5491.2012.03605.x PMC3433794

[18] Aujoulat I, Jacquemin P, Rietzschel E, et al.: Factors associated with clinical inertia: an integrative review. Adv Med Educ Pract 2014;5:141–147 24868181 10.2147/AMEP.S59022 PMC4028485

[19] Anhalt H, Bohannon NJV: Insulin patch pumps: their development and future in closed-loop systems. Diabetes Technol Ther 2010;12(Suppl 1):S51–S58 20515308 10.1089/dia.2010.0016 PMC2924780

[20] Dreon DM, Hannon TM, Cross B, et al.: Laboratory and benchtop performance of a mealtime insulin delivery system. J Diabetes Sci Technol 2018;12:817–882 29488399 10.1177/1932296818760633 PMC6134303

[21] Bergenstal RM, Johnson M, Powers MA, et al.: Adjust to target in type 2 diabetes: comparison of a simple algorithm with carbohydrate counting for adjustment of mealtime insulin glulisine. Diabetes Care 2008;31:1305–1310 18364392 10.2337/dc07-2137 PMC2453649

[22] Johnson ML, Dreon DM, Levy BL, Bergenstal RM: Insulin titration algorithms incorporated into a patient glucose diary result in significant improvements in glucose profiles and A1C. Diabetes 2018;67(Suppl 1):A186; Poster 710-P

[23] Seaquist ER, Anderson J, Childs B, et al.: Hypoglycemia and diabetes: a report of a workgroup of the American Diabetes Association and the Endocrine Society. Diabetes Care 2013;36:1384–1395 23589542 10.2337/dc12-2480 PMC3631867

[24] Gonzalez JS, Schneider HE, Wexler DJ, et al.: Validity of medication adherence self-reports in adults with type 2 diabetes. Diabetes Care 2013;36:831–837 23204245 10.2337/dc12-0410 PMC3609536

[25] Wilson IB, Fowler FJ, Cosenza CA, et al.: Cognitive and field testing of a new set of medication adherence self-report items for HIV care. AIDS Behav 2014;18:2349–2358 24077970 10.1007/s10461-013-0610-1 PMC4000749

[26] Bott U, Mühlhauser I, Overmann H, Berger M: Validation of a diabetes-specific quality-of-life scale for patients with type 1 diabetes. Diabetes Care 1998;21:757–769 9589237 10.2337/diacare.21.5.757

[27] Center for Devices and Radiological Health (CDRH): Guidance for Industry and Food and Drug Administration Staff: The Content of Investigational Device Exemption (IDE) and Premarket Approval (PMA) Applications for Artificial Pancreas Device System. U.S. Department of Health and Human Services. Food and Drug Administration. Center for Devices and Radiological Health (CDRH). November 9, 2012 https://www.fda.gov/downloads/medicaldevices/deviceregulationandguidance/guidancedocuments/ucm259305.pdf (accessed 14, 2018)

[28] Bohannon N, Bergenstal R, Cuddihy R, et al.: Comparison of a novel insulin bolus-patch with pen/syringe injection to deliver mealtime insulin for efficacy, preference, and quality of life in adults with diabetes: a randomized, crossover, multicenter study. Diabetes Technol Ther 2011;13:1031–1037 21732797 10.1089/dia.2011.0047 PMC4346544

[29] Peyrot M, Rubin RR, Chen X, Frias JP: Associations between improved glucose control and patient reported outcomes after initiation of insulin pump therapy in patients with type 2 diabetes mellitus. Diabetes Technol Ther 2011;13:471–476 21355725 10.1089/dia.2010.0167

[30] Ceriello A: Postprandial hyperglycemia and diabetes complications: is it time to treat? Diabetes 2005;514:1–7 10.2337/diabetes.54.1.1 15616004

[31] Monnier L, Mas E, Ginet C, et al.: Activation of oxidative stress by acute glucose fluctuations compared with sustained chronic hyperglycemia in patients with type 2 diabetes. JAMA 2006;295:1681–1687 16609090 10.1001/jama.295.14.1681

[32] Node K, Inoue T: Postprandial hyperglycemia as an etiological factor in vascular failure. Cardiovasc Diabetol 2009;8:23 19402896 10.1186/1475-2840-8-23 PMC2688503

[33] Bradley C, Eschwege E, dePablos-Velasco P, et al.: Predictors of quality of life and other patient-reported outcomes in the PANORAMA multinational study of people with type 2 diabetes. Diabetes Care 2018;41:267–276 29183910 10.2337/dc16-2655

[34] Edelman SV, Polonsky WH: Type 2 diabetes in the real world: the elusive nature of glycemic control. Diabetes Care 2017;40:1425–1432 28801473 10.2337/dc16-1974

